# Artificial intelligence versus classical scoring systems: a comparative analysis of stone-free prediction after percutaneous nephrolithotomy

**DOI:** 10.1007/s00240-026-01946-x

**Published:** 2026-01-30

**Authors:** Burak Elmaağaç, Ali Yasin Özercan, Abdullah Gölbaşı, Hüseyin Biçer, Ercan Arslan, Mert Ali Karadağ

**Affiliations:** 1 Kayseri Faculty of Medicine, Department of Urology, Health Sciences University, Kayseri, Türkiye; 2 Department of Urology, Kayseri City Hospital, Kayseri, Türkiye

**Keywords:** Percutaneous nephrolithotomy, Stone-free rate, Artificial intelligence, ChatGPT, Guy’s stone score, S.T.O.N.E., Machine learning, Urolithiasis

## Abstract

This study aimed to compare the predictive performance of traditional stone scoring systems with a large language model based on ChatGPT in estimating stone-free rates following percutaneous nephrolithotomy. A total of 340 patients who underwent the procedure between 2019 and 2025 were retrospectively analyzed. Preoperative stone complexity was evaluated using four established scoring systems—Guy’s Stone Score, the CROES nomogram, the S.T.O.N.E. nephrolithometry score, and the Seoul National University Renal Stone Complexity score—and each case was additionally processed through a ChatGPT-based prediction model. The predicted outcomes of each method were compared with actual postoperative results using correlation analysis and multivariate regression. The overall stone-free rate was 60.9%. Patients who achieved stone-free status had significantly lower Guy’s Stone Score, S.T.O.N.E., and S-ReSC values than those with residual stones (all *p* < 0.001). In contrast, neither the CROES nomogram (*p* = 0.19) nor the ChatGPT-based predicted stone-free probability (*p* = 0.549) differed significantly between the two groups. Univariate analysis revealed that higher values in Guy’s Stone Score, S.T.O.N.E., and S-ReSC scores were associated with stone-free failure. Multivariate analysis identified Guy’s Stone Score and S.T.O.N.E. score as independent predictors of surgical success. In contrast, the ChatGPT-based model showed limited predictive performance and failed to provide reliable estimates for stone-free rates in our study. These findings support the continued clinical utility of conventional scoring systems while emphasizing the need for further development and validation of artificial intelligence models. Large language models must be trained on structured clinical datasets and externally validated before their integration into surgical decision-making processes in endourology.

## Highlights

GSS, S.T.O.N.E., and S-ReSC scores predict SFR better than CROES and AI.

ChatGPT-based model showed weak accuracy in predicting stone-free status.

GSS and S.T.O.N.E. were independent predictors in multivariate analysis.

CROES nomogram and ChatGPT were not statistically significant predictors.

AI tools need refinement and validation for endourologic outcome prediction.

## Introduction

Urinary stone disease is a common urological condition with a substantial global health burden and a high recurrence rate. Percutaneous nephrolithotomy (PNL) remains the treatment of choice for large and complex renal calculi (> 20 mm). Achieving a high stone-free rate (SFR), which is considered the primary indicator of surgical success, is essential for optimizing patient outcomes and minimizing the need for secondary interventions. However, SFR after PNL may vary considerably depending on stone burden, anatomical complexity, and patient-related factors [1].

Stone-free rates following percutaneous nephrolithotomy (PNL) vary widely, particularly in patients with complex stone burdens such as staghorn calculi or unfavorable calyceal anatomy [1, 2]. To support preoperative risk stratification, several nephrolithometric scoring systems—including the Guy’s Stone Score (GSS), the Clinical Research Office of the Endourological Society (CROES) nomogram, the S.T.O.N.E. nephrolithometry score, and the Seoul National University Renal Stone Complexity (S-ReSC) score—have been developed to quantify anatomical and clinical complexity, with prior studies demonstrating broadly comparable predictive performance [2, 3]. Accurate preoperative prediction of stone-free status is therefore critical for surgical planning, patient counseling, and expectation management.

In parallel, artificial intelligence (AI) and large language models (LLMs) have gained increasing attention in medicine for clinical decision support. While AI-based tools such as ChatGPT have shown potential in medical information synthesis and patient guidance, their role in predicting surgical outcomes remains insufficiently explored [4].

The present study compares stone-free rate predictions generated by a ChatGPT-based model with those obtained from established nephrolithometric scoring systems, aiming to assess the current clinical applicability of large language models in endourological outcome prediction.

## Materials and methods

### Study design and patient selection

This retrospective cohort study was approved by the Non-Interventional Clinical Research Ethics Committee of Kayseri City Hospital (Decision No: 446, dated May 13, 2025). Patients who underwent PNL at our clinic between 2019 and 2025 and had sufficient clinical and radiological data were evaluated for inclusion. Of the 382 patients initially reviewed, 42 were excluded due to bilateral stone surgeries or incomplete records, resulting in a final cohort of 340 patients.

### Data collection and evaluated variables

Demographic and clinical data were collected retrospectively from patient files and the hospital information management system. Evaluated variables included age, sex, body mass index (BMI), comorbidities (e.g., diabetes mellitus, hypertension), stone characteristics (size, location, number, and type), prior stone surgery history, and preoperative laboratory results.

Perioperative variables comprised operation duration, number of access tracts, use of auxiliary instruments, intraoperative complications, and postoperative drainage methods.

Postoperative outcomes were assessed based on stone-free status, length of hospital stay, and the need for additional intervention. Stone-free status was determined using low-dose computed tomography (CT) or ultrasonography within the first postoperative month. Residual fragments ≤ 4 mm were considered clinically insignificant.

### Scoring systems

In this study, four commonly used scoring systems for predicting stone-free rates in the preoperative period were evaluated:

- **Guy’s Stone Score (GSS)**: This model classifies stone complexity on a four-grade scale (Grade I–IV) by assessing stone location, number of stones, and renal anatomy [5].
- **Clinical Research Office of the Endourological Society (CROES) nomogram**: This tool provides a numerical estimation of stone-free probability based on variables such as stone burden, patient age, prior surgical history, stone location, and institutional experience [6].
- **S.T.O.N.E. score**: This system incorporates five key parameters—stone size (Size), tract length (Tract), presence of obstruction (Obstruction), number of involved calyces (Number), and stone density (Essence)—to evaluate stone complexity [7].
- **Seoul National University Renal Stone Complexity (S-ReSC) score**: Developed by Seoul National University, this score estimates complexity solely based on stone location. Higher scores reflect greater anatomical complexity and are associated with lower stone-free rates [8].

Each scoring system was independently calculated for every patient using preoperative radiological images and clinical data. All calculations were standardized and performed by the same team member in accordance with the original methodology described in the literature.

### Use of ChatGPT-based urinary stone-free predictor

In addition to conventional scoring systems, this study evaluated the performance of a ChatGPT-based tool—**the Urinary Stone-Free Predictor**—in estimating postoperative stone-free rates following PNL [9]. Developed by Lombardo et al., the application functions as a clinical decision support system based on a LLM [4].

The tool integrates a variety of clinical and radiological variables to generate personalized stone-free rate predictions on a per-patient basis. Users are prompted to manually enter the following parameters:

- **Demographic data**: age, sex, BMI.
- **Comorbidities**: diabetes mellitus, hypertension.
- **Stone characteristics**: number of stones, largest diameter (mm), anatomical location (e.g., renal pelvis, lower calyx), and staghorn presence.
- **Preoperative lab data**: hemoglobin (g/dL), creatinine (mg/dL), urine culture.
- **Interventional history**: previous stone surgery, ureteral stenting, preoperative drainage.

The entered data are processed by the tool, which evaluates the provided variables within its internal framework and generates individualized estimates for stone-free outcomes across different endourological stone treatment modalities, including PNL and retrograde intrarenal surgery. The output is presented as: “Expected stone-free rate: X%.” In some cases, the model also provides brief qualitative feedback highlighting key influencing factors, reflecting its capacity for both quantitative and interpretive output.

For this study, all patient data were anonymized prior to analysis and manually entered into the Urinary Stone-Free Predictor using the predefined input fields provided by the application interface. All data were entered by a single investigator, and accuracy and consistency were ensured through cross-validation by two additional investigators.

The Urinary Stone-Free Predictor was used as an existing, externally developed clinical decision support tool to generate individualized stone-free rate estimates and was evaluated as a potential adjunct to traditional nephrolithometric scoring systems. Importantly, despite its clinical usability, the internal training dataset, algorithmic architecture, feature weighting strategy, and validation methodology of the ChatGPT-based Urinary Stone-Free Predictor have not been publicly disclosed by its developers. As such, the model does not represent a transparent, structured machine learning algorithm trained on a clearly defined surgical outcome dataset, but rather functions as a general-purpose large language model application that generates probabilistic outputs based on predefined clinical inputs. This non-transparent, “black-box” nature limits reproducibility, independent verification, and external validation of the model’s predictions.

Accordingly, its predictions were directly compared with conventional nephrolithometric scores in the same patient cohort using the same stone-free outcome definition and receiver operating characteristic (ROC) curve analysis, including the area under the ROC curve (AUC).

### TRIPOD-LLM compliance statement

This study evaluated the clinical performance of a LLM-based decision support tool—the Urinary Stone-Free Predictor. To ensure transparency and methodological rigor, the study was designed and reported in accordance with the TRIPOD-LLM guidelines (Transparent Reporting of a multivariable prediction model for Individual Prognosis Or Diagnosis – Large Language Models), which reflect current standards for AI-driven predictive modeling.

Specifically, we adhered to the TRIPOD-LLM checklist in structuring the study’s methodology, including data sources, the process for generating model outputs, prompt formulation, evaluation metrics (e.g., AUC, correlation analyses), human oversight procedures, and statistical analyses [10].

### Evaluation criteria and statistical analysis

The predictive stone-free rates generated by the scoring systems (GSS, CROES, S.T.O.N.E., and S-ReSC) and the ChatGPT-based Urinary Stone-Free Predictor were compared with the actual postoperative outcomes.

Additional analyses included Spearman correlation and logistic regression to evaluate associations between predicted and observed outcomes.

Continuous variables were reported as mean ± standard deviation or median, while categorical variables were expressed as percentages. All statistical analyses were conducted using SPSS Statistics version 20.0 (Chicago, IL), with a p-value < 0.05 considered statistically significant.

## Results

A total of 340 patients were included in the study. Postoperative stone-free status was achieved in 207 patients (60.9%), while residual stones were detected in 133 patients (39.1%).

GSS, S.T.O.N.E. score, and S-ReSC scores were found to be statistically significantly higher in the stone-bearing group compared to the stone-free group (all *p* < 0.001). In contrast, no significant difference was found between the residual-stone and stone-free groups in terms of the CROES score (*p* = 0.19). The stone-free percentages predicted by ChatGPT were similar between the two groups and did not show a statistically significant difference (*p* = 0.549) (Table 1).

A weak but statistically significant negative correlation was found between ChatGPT outputs and GSS (*r* = − 0.124; *p* = 0.022) and S.T.O.N.E. score (*r* = − 0.111; *p* = 0.041), No significant correlation was observed between CROES and S-ReSC scores and ChatGPT outputs. In the analysis between the scoring systems themselves, statistically significant positive correlations were found between GSS and S.T.O.N.E. and S-ReSC, as well as between S.T.O.N.E. and S-ReSC. The correlation analysis between the stone scoring systems and the stone-free percentages predicted by ChatGPT is presented in Table 2.

**Table 1:** In groups with and without postoperative stone-free status, stone scores and chatgpt’s stone-free percentage values, along with the comparison results

	With Stone (*n* = 133)	Stone-free (*n* = 207)	Total (*n* = 340)	*p* value
**GSS**	1.78 ± 1.07 (1)	1.22 ± 0.52 (1)	1.44 ± 0.83 (1)	0.000
**CROES**	160 ± 60.7 (155)	173.3 ± 61.8 (160)	168.1 ± 61.6 (160)	0.19
**S.T.O.N.E.**	8.41 ± 1.9 (8)	7.58 ± 1.2 (8)	7.9 ± 1.6 (8)	**0.000**
**S-ReSC**	3.86 ± 1.7 (4)	2.92 ± 1.8 (3)	3.3 ± 1.8 (3)	**0.000**
**ChatGPT**	78.9 ± 10.7 (79)	79.5 ± 11 (79)	79.2 ± 10.9 (79)	0.549

**GSS**: Guy’s Stone Score, **CROES**: Clinical Research Office of the Endourological Society, **S-ReSC**: Seoul National University Renal Stone Complexity

Bold text indicates statistical significance

**Table 2:** The correlation between stone scores and chatgpt’s stone-free percentages

		ChatGPT	GSS	CROES	S.T.O.*N*.E	S-ReSC
ChatGPT	*r* *p*	–	-0.124 0.022	0.096 0.078	-0.111 0.041	-0.09 0.098
**GSS**	**r** **p**	-0.124 **0.022**	–	-0.016 0.775	0.186 **0.001**	0.408 **0.000**
**CROES**	**r** **p**	0.096 0.078	-0.016 0.775	–	-0.160 **0.003**	-0.143 **0.008**
**S.T.O.N.E.**	**r** **p**	-0.111 **0.041**	0.186 **0.001**	-0.160 **0.003**	–	0.424 **0.000**
**S-ReSC**	**r** **p**	-0.09 0.098	0.408 **0.000**	-0.143 **0.008**	0.424 **0.000**	–

**GSS**: Guy’s Stone Score, **CROES**: Clinical Research Office of the Endourological Society, **S-ReSC**: Seoul National University Renal Stone Complexity

Bold text indicates statistical significance

In logistic regression analysis, univariate analysis showed that GSS (OR 0.409; 95% CI 0.298–0.561; *p* < 0.001), S.T.O.N.E. (OR 0.694; 95% CI 0.592–0.812; *p* < 0.001), and S-ReSC (OR 0.744; 95% CI 0.655–0.845; *p* < 0.001) were significantly associated with lower likehood of stone-free status, whereas ChatGPT (OR 1.005; 95% CI 0.985–1.025; *p* = 0.647) and CROES (OR 1.004; 95% CI 0.999–1.007; *p* = 0.054) were not. In multivariate analysis, only GSS (OR 0.457; 95% CI 0.328–0.636; *p* < 0.001) and S.T.O.N.E. score (OR 0.766; 95% CI 0.647–0.906; *p* = 0.002) remained independently associated with a reduced likelihood of stone-free status (Table 3).

**Table 3:** Results of logistic regression analysis of scores and ChatGPT in predicting stone-free status

	Univariate			Multivariate		
	OR	95% CI	*p*	OR	95% CI	*p*
**ChatGPT**	1.005	0.985–1.025	0.647			
**GSS**	0.409	0.298–0.561	**0.000**	0.457	0.328–0.636	**0.000**
**CROES**	1.004	0.999–1.007	0.054			
**S.T.O.N.E.**	0.694	0.592–0.812	**0.000**	0.766	0.647–0.906	**0.002**
**S-ReSC**	0.744	0.655–0.845	**0.000**			

**GSS**: Guy’s Stone Score, **CROES**: Clinical Research Office of the Endourological Society, **S-ReSC**: Seoul National University Renal Stone Complexity, **OR**: Odd’s Ratio, **CI**: Confidence Interval

Bold text indicates statistical significance

**Fig. 1:**
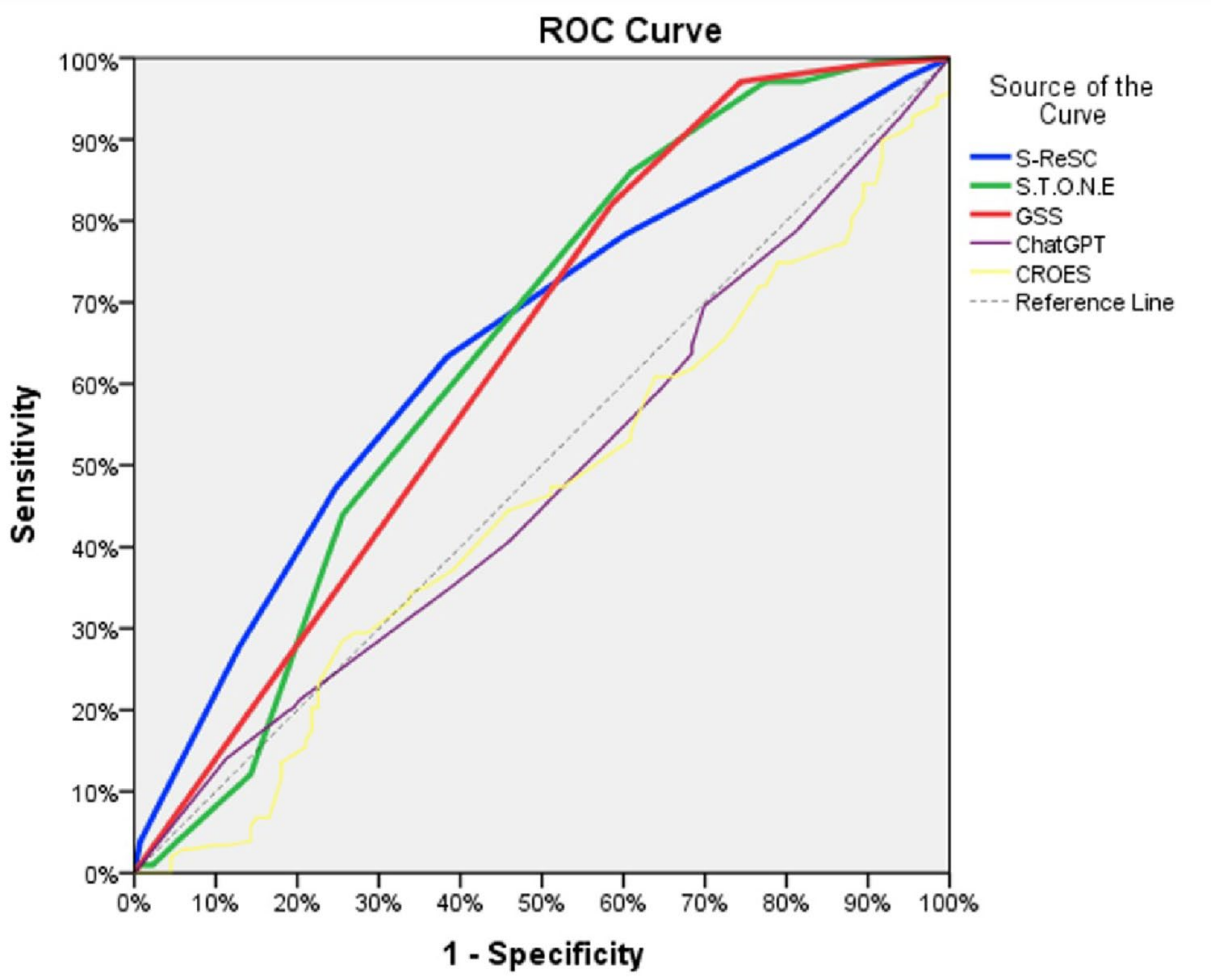
Receiver operating characteristic (ROC) curves illustrating the diagnostic performance of conventional scoring systems and a ChatGPT-based large language model for predicting stone-free status

ROC analysis demonstrated statistically significant discriminatory performance for GSS (AUC = 0.635; 95% CI 0.572–0.698), S.T.O.N.E. (AUC = 0.643; 95% CI 0.580–0.707), and S-ReSC (AUC = 0.648; 95% CI 0.589–0.708) (all *p* < 0.001). In contrast, CROES (AUC = 0.458; 95% CI 0.395–0.520; *p* = 0.19) and the ChatGPT-based model (AUC = 0.481; 95% CI 0.418–0.543; *p* = 0.552) did not show significant discriminatory performance for predicting stone-free status (Table 4). For the significant scores, optimal cut-offs were 1.5 for GSS (sensitivity 0.821, specificity 0.414), 8.5 for S.T.O.N.E. (sensitivity 0.86, specificity 0.391), and 3.5 for S-ReSC (sensitivity 0.633, specificity 0.617). ROC curves are presented in Fig. 1.

**Table 4:** ROC analysis results

	GSS	CROES	S.T.O.*N*.E	S-ReSC	ChatGPT
**AUC**	0.635	0.458	0.643	0.648	0.481
**95% CI**	0.572–0.698	0.395–0.52	0.58–0.707	0.589–0.708	0.418–0.543
**Cut-off**	1.5	–	8.5	3.5	–
**P value**	**< 0.001**	0.19	**< 0.001**	**< 0.001**	0.552
**Sensitivity**	0.821	**–**	0.86	0.633	–
**Spesifity**	0.414	**–**	0.391	0.617	–

**GSS**: Guy’s Stone Score, **CROES**: Clinical Research Office of the Endourological Society, **S-ReSC**: Seoul National University Renal Stone Complexity

Bold text indicates statistical significance

Overall, GSS and S.T.O.N.E. scores emerged as independent and significant predictors of stone-free status, whereas the ChatGPT-based model did not provide a significant contribution to stone-free prediction.

## Discussion

PNL remains the most effective treatment modality for managing large and complex kidney stones, offering higher stone-free rates compared to other minimally invasive techniques. However, achieving stone-free status depends on several variables, including stone burden, anatomical location, density, patient-specific variations, and surgeon experience [2, 11].

In our study, the observed stone-free rate (60.9%) showed close agreement with estimates derived from GSS, the S.T.O.N.E. score, and the S-ReSC system, whereas CROES and the ChatGPT-based model did not perform well in predicting stone-free status. By comparing four nephrolithometric scores alongside an AI-based tool, we were able to evaluate the relative strengths and clinical utility of each approach, supporting the continued relevance of established scoring systems in contemporary practice.

A systematic review and meta-analysis by Jiang et al. found no significant difference in the predictive performance of the GSS, the CROES nomogram, and the S.T.O.N.E. score for estimating SFR after PNL. These three models demonstrated comparable levels of accuracy. Interestingly, only GSS was found to be significantly associated with postoperative complication risk in the same review [12].

Nonetheless, some individual studies within the meta-analysis reported differing results. For instance, both GSS and S.T.O.N.E. scores were identified as significant predictors of SFR, while the CROES nomogram exhibited limited predictive value. Choi et al. observed that only GSS correlated significantly with outcomes, whereas CROES and S.T.O.N.E. did not show predictive relevance [13]. Kocaaslan et al. noted that CROES was effective only in patients with anatomical anomalies and lacked generalizability to broader populations [14]. In a study focusing on staghorn stones, Sfoungaristos et al. found that only the S.T.O.N.E. score was significantly associated with SFR, while both GSS and CROES failed to demonstrate predictive utility [15].

Similarly, in our study, both the GSS and S.T.O.N.E. scores were significantly associated with stone-free status. In contrast, the CROES nomogram did not emerge as an independent predictor in multivariate analysis. These results suggest that the predictive capacity of the CROES model may be limited, particularly in patient subgroups with complex stone morphologies.

The CROES nomogram was developed to estimate PNL success (approximately 30–90%) based on a composite of variables such as stone burden, prior stone treatment, staghorn presence, stone location/count, and the center’s annual case volume; however, its performance may vary across cohorts because it does not incorporate key radiologic determinants (e.g., hydronephrosis and other pelvicalyceal abnormalities) or stone density/composition and is relatively complex to calculate [16]. External validations have reported heterogeneous discrimination, with modest performance in a single-center cohort (AUC 0.47) but better discrimination in selected settings such as supine mini-PNL (AUC 0.695), suggesting that context and case-mix can substantially influence its utility [16, 17]. Consistent with this variability, in our cohort CROES did not differ significantly between stone-free and residual-stone groups (*p* = 0.19), showed no meaningful discriminatory performance (AUC 0.458; *p* = 0.19), and was not significant in regression analyses, supporting limited incremental value in complex referral populations.

Talyshinskii et al. recently evaluated ChatGPT’s decision-making performance in the management of urolithiasis, as well as its adherence to the EAU 2024 guidelines [18]. While the model demonstrated reasonable alignment with standard protocols for diagnosis and emergency treatment, it showed notable limitations in areas such as metabolic evaluation, prophylactic strategies, and surgical planning. These findings underscore the potential of AI-based tools for clinical information support but highlight their current limitations as standalone decision-making systems.

In our study—characterized by higher clinical complexity and referral center dynamics—there was a clear discrepancy between the actual stone-free rate (60.9%) and the model-predicted rate (79.5%). Although the ChatGPT-based predictor integrates multiple variables, including stone size, location, staghorn presence, hemoglobin levels, and BMI, its reliability remains questionable in real-world clinical settings. This is largely due to its dependence on retrospective data and the absence of external multi-center validation. The fact that GSS and S.T.O.N.E. scores retained predictive significance in our study reinforces the continued clinical value of traditional scoring systems.

Although various stone scoring systems (GSS, S.T.O.N.E., CROES, and S-ReSC) have demonstrated predictive accuracy for stone-free outcomes after PNL, their utility in forecasting complications remains limited. A comprehensive systematic review by Mazzon et al. (2023) evaluated 18 studies and concluded that these scoring tools offer little value in anticipating adverse events. Most studies reported no significant association between scoring systems and major postoperative complications such as hemorrhage, sepsis, or severe infection [19].

Moreover, many of the newly proposed nomograms were derived from retrospective, single-center cohorts and lacked external validation. These limitations underscore the need for more robust predictive models, ideally powered by artificial intelligence and supported by large-scale, multi-institutional datasets, particularly for complication risk estimation.

AI-driven tools have generated growing interest in the prediction of stone-free rates following PNL. However, the current literature reveals significant variability in their performance. For example, the ChatGPT-based “Urinary Stone-Free Predictor” developed by Lombardo et al. exhibited context-dependent accuracy despite its user-friendly design [4].

The model achieved a meaningful predictive value in ureteroscopic lithotripsy cases, with an AUC of 0.61 (*p* < 0.05), delivering useful clinical estimates within the 40–65% prediction range. In contrast, its performance for PNL patients was markedly poorer, yielding an AUC of only 0.50—comparable to random chance [4]. Consistent with this observation, in our PNL cohort the ChatGPT-based model did not demonstrate significant discriminatory performance (AUC = 0.481; *p* = 0.552), suggesting that general-purpose LLM outputs may not reliably discriminate stone-free status in complex PNL populations. Notably, classical nephrolithometric scores in our cohort also showed only modest discrimination (AUCs approximately 0.63–0.65), underscoring the inherent difficulty of stone-free prediction in high-complexity PNL case-mix and reinforcing the need for disease-specific training, formal calibration, and robust external validation when developing AI-assisted prediction tools for surgical outcomes.

In contrast to generalized large language models, customized machine learning algorithms offer improved accuracy and potentially greater generalizability when trained on disease-specific datasets. For example, Aminsharifi et al. developed a support vector machine-based model that outperformed traditional scoring systems such as GSS and CROES, achieving an AUC of 0.915 [11]. Similarly, Geraghty et al. used a large registry dataset (BAUS) to train random forest and gradient boosting models, reporting superior predictive performance for stone-free outcomes and complication risk, with an AUC of 0.76 for stone-free prediction [20]. Nevertheless, differences in case-mix, imaging protocols, and outcome definitions should be considered when comparing model performance across studies, and external validation remains essential before clinical adoption.

Among newer approaches, radiomics-based models have shown promise by leveraging high-dimensional imaging features. Zou et al. extracted more than 1,400 radiomic variables from CT images and achieved an AUC of 0.93 using a random forest algorithm, suggesting that radiomics-assisted machine learning may provide effective decision support in stone disease management [21]. However, radiomics pipelines require careful standardization, robust validation, and transparent reporting to ensure reproducibility and clinical applicability.

In our cohort, the lower predictive performance of the ChatGPT-based model may be related to its reliance on a large language model framework rather than a disease-specific predictive algorithm. Classical nephrolithometric scores are developed through statistically driven feature selection and model calibration, thereby retaining variables with independent predictive value; in contrast, prediction frameworks that aggregate numerous clinical inputs without rigorous feature selection, appropriate weighting, and formal calibration may be more vulnerable to noise and reduced discrimination—particularly when inputs include variables that have not shown consistent predictive relevance in prior validated models. In addition, limited transparency regarding training data and internal mechanisms, together with the absence of external validation, restricts the interpretability and reproducibility of language model–based tools for high-stakes clinical prediction; therefore, our findings should be viewed not as a limitation of artificial intelligence per se, but as a reflection of current constraints when applying general-purpose language models to complex surgical outcome prediction. Overall, while large language model–based predictors may currently fall short of consistently reliable accuracy for urolithiasis outcomes, disease-specific machine learning approaches trained and validated on structured clinical datasets appear more promising for forecasting stone-free status and postoperative complications following PNL.

Strengths of our study include its relatively large patient cohort, the parallel evaluation of classical scoring systems and modern AI-based models, and the generation of findings applicable to real-world clinical practice. Nonetheless, several limitations should be acknowledged. The retrospective, single-center design and the inclusion of complex referral cases may limit the generalizability of our findings to broader patient populations and different practice settings. In addition, the evolving nature of the ChatGPT algorithm may have introduced variability in predictive performance.

## Conclusion

This study shows that classical nephrolithometric scoring systems remain clinically useful for predicting stone-free outcomes after PNL in complex stone disease. In our cohort, GSS and S.T.O.N.E. emerged as independent predictors of stone-free status, while S-ReSC was associated with outcomes but did not retain independent significance in multivariable analysis. In contrast, the CROES nomogram demonstrated limited incremental utility in this referral, high-complexity setting.

General-purpose LLM outputs, exemplified by the ChatGPT-based Urinary Stone-Free Predictor, did not demonstrate meaningful discriminatory performance for stone-free prediction in our PNL population, supporting the need for disease-specific training, calibration, and robust external validation before considering LLM-based tools as standalone decision support.

Overall, while established scoring systems continue to provide practical risk stratification, future AI adoption in routine stone surgery will likely depend on well-validated, condition-specific machine learning approaches and prospective, multicenter real-world evaluation.

## Data Availability

The datasets used and analyzed during the current study are available from the corresponding author on reasonable request.
